# Reiseimpfungen bei rheumatischen Erkrankungen: Besonderheiten bei Kindern und Erwachsenen

**DOI:** 10.1007/s00393-020-00852-w

**Published:** 2020-08-26

**Authors:** T. Welzel, A. Wörner, U. Heininger

**Affiliations:** 1 Pädiatrische Rheumatologie, Universitäts-Kinderspital beider Basel (UKBB), Universität Basel, Spitalstr. 33, CH-4056 Basel, Schweiz; 2 Pädiatrische Pharmakologie, Universitäts-Kinderspital beider Basel (UKBB), Universität Basel, Spitalstr. 33, CH-4056 Basel, Schweiz; 3 Pädiatrische Infektiologie, Universitäts-Kinderspital beider Basel (UKBB), Universität Basel, Spitalstr. 33, CH-4056 Basel, Schweiz; 4 Medizinische Fakultät, Universität Basel, Basel, Schweiz

**Keywords:** Immunsuppression, Lebendimpfstoff, Totimpfstoff, Infektion, Impfschutz, Immunosuppression, Live vaccine, Inactivated vaccine, Infection, Vaccination protection

## Abstract

Kinder und Erwachsene mit rheumatischen Erkrankungen (RE) haben nicht nur durch ihre Grunderkrankung, sondern auch durch die vielfach notwendige immunsuppressive Therapie (IT) ein erhöhtes Risiko, an bestimmten Infektionen zu erkranken. Durch die IT hat sich die Lebensqualität bei vielen Patienten mit RE verbessert, sodass ihr internationales Reiseverhalten dem gesunder Reisenden ähnelt. Eine Untersuchung deutet an, dass Patienten mit Immunsuppression oftmals unzureichend auf Reisen vorbereitet sind und ihr Impfschutz schlechter als bei Immungesunden ist. Da auch während und nach Reisen das Erkrankungsrisiko für allgemeine und reisespezifische Infektionen bei Patienten mit Immunsuppression erhöht ist, sind reisemedizinische Beratungen bei Patienten mit RE wichtig. Hier können ein Reiserücktritt oder Reisemodifikationen und spezifischer Schutz inklusive Reiseimpfungen besprochen werden. Zu den gängigen Indikationsimpfungen bei Reisen zählen Impfungen gegen Hepatitis A, Typhus, Tollwut, Meningokokken, FSME (Frühsommer-Meningoenzephalitis), saisonale Influenza, Japanische Enzephalitis, Cholera, Poliomyelitis und Gelbfieber. Bei Patienten mit RE ergibt sich die Impfindikation dabei aus der möglichen Exposition gegenüber impfpräventablen Infektionen, dem individuellen Reiseverhalten, der Schwere der möglichen Infektion unter Abwägung der Risiken, die mit der Reiseimpfung assoziiert sind. Dafür müssen auch der allgemeine Gesundheitszustand, die Aktivität und Schwere der RE und der Grad der IT beachtet werden. Allgemein gilt für Patienten mit RE und IT, dass auch bei Reiseimpfungen Lebendimpfstoffe kontraindiziert sind, während Totimpfstoffe verabreicht werden können. Da eine reduzierte Impfantwort möglich ist, können Antikörpermessungen nach der Impfserie und spezifische Impfschemata oder zusätzliche Booster bei Patienten mit RE indiziert sein.

## Abstract (en)

Children and adults with rheumatic diseases (RD) have a higher risk to contract infections due to the underlying disease and the frequently necessary immunosuppressive treatment (IT). The quality of life of the majority of patients with RD has remarkably improved due to IT-related reduction of inflammation. Therefore, RD patients usually have an international travel behavior similar to healthy individuals. An investigation indicated that patients with RD and IT have lower travel vaccination rates and are often less well-prepared for their trip in comparison to healthy travelers, even when visiting high risk destinations. As the risk for general and travel-acquired infections is increased for patients with RD with and without IT, pretravel consultations are important. These pretravel consultations should include recommendations addressing travel cancellation, travel modification and travel vaccinations depending on the patient’s risk. Travel vaccinations include vaccinations against hepatitis A, typhoid fever, rabies, cholera, meningococcal diseases, tick-bone encephalitis, Japanese encephalitis, seasonal influenza, poliomyelitis and yellow fever. In patients with RD the indications for vaccination depend on the exposure risks, disease severity, individual travel behavior, and possible complications associated with vaccination. In the further evaluation process it is crucial to include the general health condition of the patient, the underlying RD (type and activity), duration and intensity of the IT. In general, live-attenuated vaccines are contraindicated under IT. In contrast, inactivated vaccines may be administered although reduced immunogenicity with the need for antibody measurement, special vaccine schedules or additional booster vaccinations should be considered under IT.

Rheumatische Erkrankungen (RE) betreffen Kinder und Erwachsene. Patienten mit RE haben ein erhöhtes Risiko, an bestimmten Infektionen zu erkranken, wobei das Infektionsrisiko von der Grunderkrankung und der individuellen immunsuppressiven Therapie (IT) abhängt [16, 20]. Auch haben immunsupprimierte Patienten ein erhöhtes Risiko, während und nach Reisen an allgemeinen und reisespezifischen Infektionen schwerer zu erkranken, als Immunkompetente [7, 13]. Dieser Beitrag bietet Hilfestellung für die reisemedizinische Impfberatung bei Patienten mit RE. Die Fachinformationen der Impfstoffe und der IT müssen bei der individuellen Patientenberatung zusätzlich beachtet werden.

## Bedeutung von Impfungen bei rheumatischen Erkrankungen

Patienten mit RE haben ein erhöhtes Infektionsrisiko für virale, bakterielle, fungale oder parasitäre Infektionen, was von der Grunderkrankung und der immunsuppressiven Therapie (IT) abhängt [16, 20]. Auch verlaufen impfpräventable Krankheiten bei Patienten mit RE und IT schwerer und komplikationsträchtiger als bei Immunkompetenten [13, 29]. Da sich die Lebensqualität von Patienten mit RE durch die IT deutlich gebessert hat, steigt die Nachfrage nach Fernreisen, und viele scheinen mittlerweile ein ähnliches Reiseverhalten wie immunkompetente Reisende zu zeigen [17]. Eine Untersuchung deutet dabei an, dass immunsupprimierte Patienten oft unzureichend auf die Reise vorbereitet sind, selbst bei Reisen in Hochrisikogebiete [3]. Da immunsupprimierte Patienten ein erhöhtes Risiko haben, während und nach Reisen an allgemeinen und reisespezifischen Infektionen zu erkranken [7, 13], sind für sie die von der STIKO (Ständige Impfkommission) empfohlenen Standard- und Indikationsimpfungen sowie Reiseimpfungen von großer Bedeutung. Für Patienten mit autoinflammatorischen Erkrankungen gelten im Wesentlichen dieselben Anwendungshinweise wie für Patienten mit autoimmunologischen RE [9].

### Totimpfstoffe

Totimpfstoffe enthalten inaktivierte Erreger oder immunogene Erregerbestandteile. Sie können bei Patienten mit RE mit und ohne IT sicher verabreicht werden [11, 12, 14, 21, 25, 29]. Für einen optimalen Impferfolg sollte die Impfung 2, besser 4 Wochen vor IT abgeschlossen sein (Rituximab ≥4 Wochen) [29]. Während der IT sollte bei stabiler Erkrankung und/oder möglichst geringer IT geimpft werden [4, 29].

Die Immunogenität der Impfung wird durch die Grunderkrankung und die IT beeinflusst und kann im Vergleich zu Gesunden reduziert sein [12, 21, 29]. Meist wird aber ein ausreichender Impfschutz erreicht, ausgenommen sind B‑ und/oder T‑Zell-depletierende Antikörpertherapien [29]. Die Kontrolle des Impferfolgs durch Messung von spezifischen Antikörpern (AK) nach abgeschlossener Impfserie mit Erreichen bzw. Überschreiten definierter Schutzkorrelate kann sinnvoll sein [21].

### Lebendimpfstoffe

Lebendimpfstoffe enthalten abgeschwächte (attenuierte), aber noch replikationsfähige Viren oder Bakterien. Bei Patienten mit RE ohne IT können Lebendimpfstoffe ohne zusätzliche (zu den allgemein in der Fachinformation erwähnten) Sicherheitsbedenken verabreicht werden; ein Mindestabstand von 4 Wochen vor IT-Beginn sollte eingehalten werden [25, 29]. Unter IT sollten allgemein keine Lebendimpfungen verabreicht werden [14, 25, 29]. Abhängig vom Schweregrad der IT können jedoch nach individueller Risiko-Nutzen-Abwägung bestimmte Lebendimpfungen erwogen werden [14, 29].

## Reisen mit rheumatischen Erkrankungen

**Figure 1:**
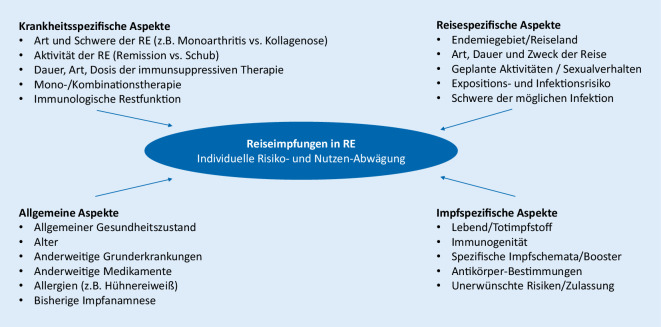

Vor Reisen sollten Personen mit RE nach den üblichen reisemedizinischen Empfehlungen (z. B. Deutsche Gesellschaft für Tropenmedizin und Internationale Gesundheit, www.dtg.org) beraten werden. Neben der Impfberatung ist die Aufklärung über allgemeine Verhaltensmaßnahmen wie Hände- und Nahrungsmittelhygiene sowie Mückenschutz essenziell. Auch sollte der Reisende beraten werden, ob ein Reiserücktritt oder eine Reisemodifikation in Anbetracht des individuellen Risikos sinnvoll ist. Welche Reiseimpfungen indiziert sind, ergibt sich aus der möglichen Exposition gegenüber impfpräventablen Infektionen, der Schwere der möglichen Infektion und den Risiken, die mit der Reiseimpfung assoziiert sind [10]. In diesen Entscheidungsprozess sollte auch das individuelle Reiseverhalten des Patienten einbezogen werden [10]. Hierzu gehören zumindest Art, Dauer und Zweck der Reise, Unterkunft und Ernährung sowie geplante Aktivitäten vor Ort und das Sexualverhalten [10]. Bei Patienten mit RE müssen zusätzlich der allgemeine Gesundheitszustand, die Aktivität und die Schwere der RE sowie der Grad der IT beachtet werden. Dabei ist es auch wichtig, die Art des Impfstoffs (Tot‑/Lebendimpfstoff) zu berücksichtigen (Abb. 1). Zusätzlich muss geprüft werden, ob mögliche Interaktionen zwischen ggf. nötiger Chemoprophylaxe (z. B. bei Malaria) und IT vorliegen.

### Ausmaß der Immunsuppression

Vor Impfung ist eine individuelle Einschätzung der aktuellen Immunkompetenz notwendig, die durch den allgemeinen Gesundheitszustand, die Aktivität und Art der RE, Dosis und Art sowie Dauer der IT beeinflusst wird [29]. Einen Anhalt zur Einordnung der immunsuppressiven Wirkung bestimmter IT, basierend auf im Wesentlichen empirischen Daten und Expertenerfahrungen, fasst Tab. 1 zusammen. Spezifische Tests können helfen, die immunologische Reaktionsfähigkeit des Patienten unter IT besser einzuordnen [26]. Folgende Bestimmungen können im Bedarfsfall vor Lebendimpfung gemäß empirischen Daten und Expertenmeinungen hilfreich sein und werden vorgeschlagen [26]:

- Lymphozytenzahl ≥1200/µl und/oder CD4(+)-T-Helferzellen ≥200/µl (ab Alter 6 Jahre) bzw. ≥500/µl (Alter 0 bis 5 Jahre), ergänzend für Patienten nach Rituximab-Therapie: B‑Zellen sollten vor Lebendimpfung wieder im Altersnormbereich sein,
- Immunglobulin G ≥(5–)7g/l im Serum,
- vorhandene Impftiter, z. B. gegen Tetanustoxin (ist dieser niedrig oder negativ, wird ein Booster unter IT mit Verlaufstiterkontrolle empfohlen).

**Table 1:** 

Schweregrad der immunsuppressiven Wirkung	Therapie
Keine oder gering	Hydroxychloroquin
	Sulfasalazin
	Mesalazin
Gering	*Niedrigdosierte systemische Glukokortikoide* – Erw.: <10 mg Prednisolonäquivalent/Tag – Kinder: <0.2 mg/kg/Tag Prednisolonäquivalent – Kurzzeittherapie (<2 Wochen)
	*Nicht systemische Glukokortikoide* – Topisch (dermal, inhalativ, konjunktival, nasal) – Intraartikulär
	*Niedrigdosierte Basistherapeutika* MTX – Erw.: ≤0.4 mg/kg/Woche oder ≤20 mg/Woche – Kinder ≤15 mg/m^2^/Woche Ciclosporin (≤2.5 mg/kg/Tag) Leflunomid – Erw.: ≤20 mg/Tag – Kinder ≤0.5 mg/kg/Tag Mycophenolat-Mofetil – Erw.: ≤2000 mg/Tag – Kinder ≤1200 mg/m^2^/Tag Tofacitinib – Erw.: ≤ 5–10 mg/Tag
	*Einige niedrigdosierte Biologika, z. B.* Infliximab (≤3 mg/kg/8 Wochen)
Schwer	*Hochdosierte systemische Glukokortikoide* – Dosierung oberhalb der oben genannten Grenzwerte – Therapiedauer ≥2 Wochen – Intravenöse Stosstherapie mit hohen Dosen (z. B. 20 mg/kg/Tag Prednisolonäquivalent über mehrere Tage monatlich wiederholt)
	*Hochdosierte Basistherapeutika * – Dosierung oberhalb der oben genannten Grenzwerte
	Azathioprin
	*Biologika mit schwerer Immunsuppressiver Wirkung, z. B.* – Infliximab (≥5 mg/kg/4 Wochen bzw. ≥7 mg/kg/8 Wochen) – Abatacept – Rituximab
	*Kombinationen von Immunsuppressiva*

*Erw*. Erwachsene, *MTX* Methotrexat

### Allgemeine und krankheitsspezifische Impfungen

Bei der Reiseimpfberatung sollten neben den klassischen Reiseimpfungen bei Patienten mit RE ggf. auch alle bisher nicht erfolgten allgemein oder krankheitsbedingt empfohlenen Impfungen unter Berücksichtigung möglicher Kontraindikationen nachgeholt werden [4] (Tab. 2). Bei sexuellem Risikoverhalten und bei Jugendlichen sollte die Impfung gegen Hepatitis B und humane Papillomviren besprochen werden, und bei unzureichender Immunisierung sollte diese empfohlen bzw. nachgeholt werden.

**Table 2:** 

Impfung gegen	Lebendimpfstoff	Totimpfstoff	Mindestalter und Kommentare
Cholera	–	x	DUKORAL® (Valneva Sweden AB, Schweden): 2 J
Frühsommer‐Meningoenzephalitis	–	x	Encepur® Kinder (GlaxoSmithKline, Vereinigtes Königreich)/FSME-Immun® 0,25 ml Junior (Pfizer Pharma, Vereinigte Staaten): 1 J. <3 J. Fieberreaktionen häufig, individuelle Indikationsstellung
Gelbfieber	x	–	STAMARIL® (Sanofi, Frankreich): 9 (6) Mo, KI: IT Zugelassen ab 6 Mo., allerdings erhöhtes Enzephalitisrisiko zwischen 6 und 9 Mo, daher strenge Risiko-Nutzen-Evaluation
Hepatitis A	–	x	Havrix®720 Kinder (GlaxoSmithKline, Vereinigtes Königreich)/VAQTA® Kinder (MSD Merck Sharp & Dohme, Vereinigte Staaten): 1 J. Havrix1440® (GlaxoSmithKline, Vereinigtes Königreich): 15 J. VAQTA® (MSD Merck Sharp & Dohme, Vereinigte Staaten): 18 J.
Hepatitis A+B	–	x	Twinrix® Kinder (GlaxoSmithKline, Vereinigtes Königreich): 1 J. Twinrix® (GlaxoSmithKline, Vereinigtes Königreich): 16 J.
Japanische Enzephalitis	–	x	IXIARO® (diverse Hersteller): 2 Mo.
Meningokokken B	–	x	Bexsero® (GlaxoSmithKline, Vereinigtes Königreich): 2 Mo. Trumenba® (Pfizer Pharma, Vereinigte Staaten): 10 J.
Meningokokken ACWY-Konjugat	–	x	Menveo® (GlaxoSmithKline, Vereinigtes Königreich): 2 J. Nimenrix® (Pfizer Pharma, Vereinigte Staaten): 6 Wochen
Poliomyelitis	–	x	IPV Merieux® (Sanofi, Frankreich) /Kombinationsimpfung (diverse Hersteller): Grundimmunisierung gemäß STIKO ab dem Alter von 2 Mo. (idealerweise als Kombinationsimpfung), Reiseindikation beachten
Tollwut	–	x	Rabipur® (GlaxoSmithKline, Vereinigtes Königreich)/Tollwutimpfstoff (HDC) inaktiviert® (Sanofi, Frankreich): ab Geburt
Typhus	x (Typhoral®)	x (Typhim Vi®)	Typhim Vi® (Sanofi, Frankreich): 2 J. Typhoral® L Kapseln (Pharma K Medical GmbH; Deutschland): 5 J., KI: IT
**Die STIKO-Standardimpfungen [** 27**] sowie krankheitsspezifische Impfungen sollten zusätzlich unter Berücksichtigung von KI bei Reisen altersgemäß aktuell sein:**			
*Diphtherie/Tetanus/Pertussis/Poliomyelitis/Haemophilus influenzae Typ b*			
*Hepatitis B*			
*Humanpathogene Papillomaviren*			
*Pneumokokken*			
*MMR* – Idealerweise vor IT (2 Dosen) – KI bei schwerer Immundefizienz/IT – Niedrigdosierte Glukokortikoide sind keine KI – Verabreichung laut Fachinformationen und Expertenkonsens im Einzelfall bei geringer Immunsuppression nach individueller Risiko-Nutzenabwägung möglich			
*Varizellen* – Idealerweise vor IT (2 Dosen) – Verabreichung laut Fachinformationen nach individueller Risiko-Nutzenabwägung in Abhängigkeit von der Immunsuppression unter Therapie/in Therapiepausen nach immunologischer Vordiagnostik möglich, wenn ein besonderes Gesundheitsrisiko vorliegt und die Gesamtlymphozytenzahl bei Erwachsenen mindestens 1200/mm^3^ Blut beträgt (bei Kindern altersabhängige Referenzwerte) und/oder kein anderer Hinweis auf unzureichende zelluläre Immunität besteht			
*Influenza (KI: nasaler Lebendimpfstoff bei IT)*			

*IT* immunsuppressive Therapie, *J.* Jahre,* KI* Kontraindikation, *Mo.* Monate*, STIKO* ständige Impfkommission, *MMR* Masern, Mumps, Röteln

#### Hepatitis B

Hepatitis B wird durch das *Hepatitis-B-Virus* ausgelöst. Die Übertragung erfolgt v. a. durch Intimkontakte, mukokutane Kontakte mit infektiösem Material, kontaminierte Bluttransfusionen und perinatal. Die Hepatitis-B-Impfung sollte entsprechend der STIKO Empfehlung erfolgen [27]. Bei Risikopersonen sollte 4 bis 8 Wochen nach der Grundimmunisierung eine Anti-HBs(Hepatitis B-Surface)-AK-Kontrolle erfolgen [29]. Abhängig vom serologischen Erfolg kann eine vierte Impfdosis indiziert bzw. je nach Ansprechen anschließend ein Impfstoffwechsel mit höherer Antigendosis oder zusätzlichem Adjuvans nötig sein [9].

#### Humanpathogene Papillomaviren

Das Risiko für HPV(humanpathogene Papillomaviren)-Infektionen ist bei Immunsupprimierten höher als bei Gesunden, besonders auch beim systemischen Lupus erythematodes [29, 30]. Die STIKO empfiehlt 9‑ bis 14-jährige Jungen und Mädchen mit 2 Impfdosen im Abstand von ≥5 Monaten zu impfen [27]. Nachholimpfungen für ältere Personen beinhalten eine dritte Impfdosis [27]. Da die AK-Spiegel bei Immunsupprimierten im Vergleich zu Gesunden reduziert sein können, können diese altersunabhängig von einem 3‑Dosen-Impfschema profitieren [29]. Die vollständige Impfserie sollte vor dem ersten Sexualkontakt abgeschlossen sein [27].

### Reiseimpfungen

Bei den Reiseimpfungen wird unterschieden zwischen [8, 18]:

- Pflichtimpfungen, die zur Einreise in einzelnen Ländern vorgeschrieben sind (z. B. Gelbfieberimpfung),
- Standardimpfungen, die nach dem STIKO-Impfkalender vorliegen sollten, und
- Indikationsimpfungen, die entsprechend dem Reiserisiko angezeigt sind.

Zu den Reiseimpfungen (Tab. 2) zählen beispielsweise Hepatitis A, Typhus, Tollwut, Meningokokken, FSME (Frühsommer-Meningoenzephalitis), saisonale Influenza, Japanische Enzephalitis, Cholera und Gelbfieber [8]. Die Impfung gegen Gelbfieber, Masern-Mumps-Röteln, Varizellen sowie die orale Typhusimpfung erfolgen mit Lebendimpfstoffen. Die Gelbfieberimpfviren haben eine hohe Replikationskapazität, der orale Typhusimpfstoff hat eine geringe [4]. Allgemein sollten keine Reiseimpfungen mit Lebendimpfstoffen bei Patienten mit RE und IT erfolgen, allerdings kann nach individueller Risikoabwägung unter entsprechenden Vorsichtsmaßnahmen eine Impfindikation gestellt werden [4]. Lebendimpfungen können mit gewisser Latenz nach IT-Stopp erfolgen, dabei variieren die Zeitintervalle je nach IT und Impfung [4, 10, 13]. Reiseimpfungen mit Totimpfstoff gelten als sicher, allerdings kann der Impfschutz bei RE mit und ohne IT reduziert sein [4, 19, 29].

## Spezifische Informationen zu einzelnen Reiseimpfungen

### Cholera

Cholera wird durch Aufnahme von kontaminierten Nahrungsmitteln/Trinkwasser durch das Bakterium *Vibrio cholerae *ausgelöst. Maßnahmen zur Nahrungs-und Trinkwasserhygiene sollten beachtet werden, da diese bereits alleine hocheffektiv eine Choleraübertragung verhindern können [23]. Die Impfung kann zusätzlich bei Aufenthalt in Infektionsgebieten speziell unter mangelhaften Hygienebedingungen bei aktuellen Ausbrüchen z. B. in Flüchtlingslagern oder bei Naturkatastrophen indiziert sein [27]. Der orale Totimpfstoff kann nach Fachinformation bei Patienten mit Immunsuppression verabreicht und sollte je nach Reiseland, Reisedauer und erwarteter Exposition empfohlen werden [19, 29], allerdings sollte berücksichtigt werden, dass die Wirksamkeit bei vorübergehend Exponierten unbekannt zu sein scheint und auch kein Schutz gegen Serovar O139 besteht [23]. Die impfinduzierten antitoxischen AK im Darm können darüber hinaus einen partiellen Schutz gegen das „Cholera-like-Toxin“ der enterotoxischen *Escherichia coli *bieten [8].

### Frühsommer-Meningoenzephalitis

FSME wird durch *Flaviviren* ausgelöst und überwiegend durch die Schildzecke *Ixodes ricinus *übertragen. Die FSME-Impfung wird allen Personen empfohlen, die in Risikogebieten zeckenexponiert sind [27]. Die Impfung sollte auch bei Patienten mit RE mit und ohne IT entsprechend den STIKO-Empfehlungen erfolgen [29]. Bei Patienten mit IT kann die Impfantwort eingeschränkt sein [15].

### Gelbfieber

Gelbfieber wird durch ein *Flavivirus* verursacht. Der Hauptvektor ist die tagesaktive Mücke *Aedes aegypti. *Die Gelbfieberimpfung ist bei Immunsuppression kontraindiziert [4, 11, 19, 25, 29]. Personen mit schwerer Immunsuppression sollte von Reisen in Gelbfieberendemiegebiete abgeraten werden [19, 25, 29].

Die Gelbfieberimpfung ist bei Immunsuppression kontraindiziert

Für die Einreise in bestimmte Länder ist der Nachweis einer Impfung erforderlich. Bei medizinischer Kontraindikation und Reisenotwendigkeit kann ein „exemption certificate“ ausgestellt werden, das von einer Impfung entbindet [19, 24, 29]. Die Persistenz von neutralisierenden AK scheint bei Patienten, die vor IT gegen Gelbfieber geimpft worden sind, nicht beeinflusst [6]. Allerdings ist für eine Langzeitimmunität eine zweite Impfdosis empfohlen [6].

### Hepatitis A

Hepatitis A wird durch das *Hepatitis-A-Virus* fäkal-oral übertragen und ist eine häufige Infektionskrankheit bei Reisenden in Risikogebiete. Die Impfung wird allen, auch Immunsupprimierten, ab dem Alter von 1 Jahr bei Reisen in Regionen mit hoher Hepatitis-A-Inzidenz empfohlen [27, 29]. Studien zeigen bei Patienten mit IT im Vergleich zu Immunkompetenten, insbesondere unter Tumor-Nekrose-Faktor(TNF)-Inhibitoren, eine unzureichende Seroprotektion nach erster Impfdosis [1, 28]. Eine zusätzliche Impfdosis parallel zur ersten oder 4 Wochen nach erster Dosis kann bei IT den Impferfolg verbessern [22]. Eine dritte Dosis sollte nach 6 bis 12 Monaten verabreicht werden, um einen Langzeitschutz sicherzustellen [29].

### Japanische Enzephalitis (JE)

Die JE ist eine *Flavivirus*-Infektion, die durch die nachtaktive *Culexmücke* in Teilen von Asien und der westpazifischen Region übertragen wird [24]. Sie ist eine der häufigsten Ursachen weltweit für virale Enzephalitiden und geht mit einer Letalität von etwa 30 % sowie bei Überlebenden mit hohen Raten an Residualschäden einher [18, 24]. In Deutschland ist ein Totimpfstoff ab 2 Monaten zugelassen und wird in 2 Dosen im Abstand von 4 Wochen verabreicht. Im Alter von 2 Monaten bis <3 Jahre wird die Hälfte des Impfstoffs verabreicht, ab 3 Jahren wird die volle Dosis gegeben [24]. Bei Reisen in Endemiegebiete scheint für Patienten mit Immunsuppression, insbesondere unter Therapie mit TNF-Inhibitoren das Risiko für die JE erhöht, sodass die Impfung bei dieser Personengruppe besonders indiziert zu sein scheint [5, 29].

### Meningokokken (B, ACWY)

Die Meningokokkenmeningitis mit/ohne Sepsis wird durch invasive Stämme von *Neisseria meningitidis *hervorgerufen und kann lebensbedrohlich verlaufen. Bei der Meningokokkenimpfung muss die Epidemiologie berücksichtigt werden. Die in Deutschland verfügbaren quadrivalenten Meningokokkenkonjugatimpfstoffe schützen vor Serogruppen ACWY, aber nicht vor Serogruppe B. Es sind 2 Impfstoffe gegen B‑Meningokokken in Deutschland zugelassen [24]. Eine Impfung gegen die Serogruppe C wird in den STIKO-Standardimpfungen bis 18 Jahre empfohlen [27]. Die quadrivalente Meningokokkenimpfung sollte allen Reisenden in Endemiegebiete, z. B. mit Ziel afrikanischer „Meningitisgürtel“, empfohlen werden [18, 24, 27]. Der Meningitisgürtel zieht sich von Senegal und Gambia bis nach Äthiopien [24]. Auch können Impfindikationen bei Reisen in (sub)tropische Regionen mit niedrigem sozioökonomischem Status und engem Kontakt zu Einheimischen, bei Langzeitschüleraustausch oder Studienaufenthalt im Ausland bestehen [24]. Bei Patienten mit Immunsuppression kann die Impfantwort reduziert sein [29]. Es gibt daher z. B. in der Schweiz die Empfehlung, Patienten mit erhöhtem Risiko für invasive Meningokokkenerkrankungen im Alter von 2 bis 11 Monaten mit 4 Dosen (Zeitpunkt 2, 3, 4 und 12 Monate) und im Alter ≥12 Jahren mit 2 Dosen des quadrivalenten Impfstoffs im Abstand von 4 bis 8 Wochen zu impfen [2]. Bei fortbestehendem Infektionsrisiko sollten alle 5 Jahre Booster erfolgen [2].

### Poliomyelitis

Die Poliomyelitis wird durch fäkal-orale Infektion mit dem *Poliovirus* verursacht. Eine Grundimmunisierung einschließlich Auffrischimpfung gegen Poliomyelitis wird in den STIKO-Standardimpfungen empfohlen [27]. Sowohl beim Kombinationsimpfstoff (Diphtherie, Pertussis, Tetanus, Polio) wie auch bei dem inaktivierten Poliovakzin (IPV) handelt es ich um Totimpfstoffe, sodass auch Patienten mit IT geimpft werden können. Eine IPV-Auffrischimpfung wird bei Reisen in Regionen mit Infektionsrisiko empfohlen, wenn die letzte Impfung bei abgeschlossener Grundimmunisierung >10 Jahre zurückliegt [27]. Neben formalen Impfindikationen (Länderbestimmungen beachten) hat die WHO (Weltgesundheitsorganisation) auch für bestimmte Länder verschärfte temporäre Impfempfehlungen ausgesprochen, sodass kürzere Impfabstände gelten können (Informationen des Auswärtigen Amts beachten) [8, 27]. Bei Personen ohne Grundimmunisierung sollten vor Reisebeginn mindestens 2 IPV-Impfdosen im 4‑Wochen-Abstand verabreicht werden [27]. Bei fehlenden oder nicht dokumentierten Impfungen der Grundimmunisierung sollten diese nachgeholt werden [27].

### Tollwut

Der Tollwuterreger ist das *Rabiesvirus*, das bei einem Biss von infizierten Säugetieren übertragen werden kann. Die klassische Form der Tollwut verläuft letal. Die Tollwutimpfung ist bei Reisen in Regionen mit erhöhter Tollwutgefährdung empfohlen [27]. In Deutschland sind 2 Totimpfstoffe auf Basis inaktivierter Tollwutviren zugelassen [24]. Die präexpositionelle Tollwutimpfung (HDC[human diploid cell]- oder PCEC[purified chick embryo cell]-Impfstoff) wird nach dem in der Fachinformation genannten Impfschema (3 Impfstoffdosen Tag 0, 7, 28) empfohlen [9]. Ein Schnellimpfschema („off-label“) wird nicht empfohlen [9]. Für immundefiziente Personen ist eine AK-Bestimmung 2 bis 4 Wochen nach vollständiger Grundimmunisierung mit erneuter Impfung bei einem AK-Spiegel <0,5 IE/ml empfohlen [8, 9]. Dies Vorgehen empfehlen wir auch bei Patienten mit RE mit und ohne IT. Bei länger notwendigem Impfschutz kann durch AK-Bestimmung abgeschätzt werden, ob eine Auffrischimpfung notwendig ist und empfohlen werden sollte. Auch kann man sich an den Anwendungshinweisen für Reisende mit Immundefizienz orientieren. Hier wird eine Auffrischung 1 Jahr nach erster Impfung und dann alle 5 Jahre mit je einer Impfdosis empfohlen [9]. Kommt es bei Ungeimpften zu Kontakt mit tollwutverdächtigen Tieren (Biss‑/Kratzwunden, Speichelkontakt mit offenen Hautstellen/Schleimhäuten), muss umgehend eine Postexpositionsprophylaxe (PEP) durchgeführt werden [18].

Bei Ungeimpften mit Kontakt zu tollwutverdächtigen Tieren muss umgehend eine PEP durchgeführt werden

Bei Patienten mit Immunsuppression sollte dabei bei Exposition ab Grad II eine PEP mit 5 Impfstoffdosen nach dem Essen-Schema an Tag 0, 3, 7, 14, 28 eingeleitet werden, auch wenn eine präexpositionelle Impfserie durchgeführt wurde [9, 19]. Eine simultane Gabe von Immunglobulinen sollte erfolgen und kann bis zu 7 Tage nach Beginn der PEP nachgeholt werden [9, 19]. Die Fachinformation sollte beachtet werden. Eine Titerbestimmung sollte 7 bis 14 Tage nach der letzten Impfung im Rahmen der PEP erfolgen [9, 19].

### Typhus

Typhus abdominalis wird durch *Salmonella typhi* über infizierte Nahrungsmittel, seltener durch Schmierinfektion ausgelöst. Die Impfung sollte bei Reisen nach Südasien und in andere Endemiegebiete, bei Langzeitaufenthalten mit niedrigen Hygienestandards und bei Naturkatastrophen erfolgen [24]. Je nach Reiseland, Reisedauer und Expositionsrisiko sollten auch Patienten mit RE mit und ohne IT mit dem inaktivierten Typhusimpfstoff geimpft werden [19, 29]. Bei Risikopatienten sollte die Impfindikation großzügiger als bei Immunkompetenten gestellt werden [19].

## Fazit für die Praxis

- Patienten mit rheumatischen Erkrankungen sollten vor internationalen Reisen eine reisemedizinische Beratung erhalten, in der unter Beachtung des individuellen Infektionsrisikos Reiserücktritt, Reisemodifikationen, Schutzmaßnahmen und Reiseimpfungen besprochen werden.
- Die Indikation für eine Reiseimpfung ergibt sich aus reisespezifischen Faktoren, dem allgemeinen Gesundheitszustand, der Art und Aktivität der Grundkrankheit und Dauer, Dosis und Art der immunsuppressiven Therapie.
- Fachinformationen der Impfstoffe und immunsuppressiven Medikamente müssen beachtet werden; auch sind ggf. individuelle Risiko-Nutzen-Abwägungen wichtig.
- Reiseimpfungen mit Totimpfstoffen können bedenkenlos erfolgen, wohingegen Lebendimpfstoffe (z. B. Gelbfieberimpfung) allgemein unter immunsuppressiver Therapie kontraindiziert sind.
- Der Impferfolg, beeinflusst durch Grunderkrankung und Therapie, kann reduziert sein, daher sollten Antikörperbestimmungen 4 bis 8 Wochen nach abgeschlossener Impfserie erwogen werden.
- Zusätzliche Impfdosen können für den optimierten Impfschutz nötig sein (z. B. Hepatitis A).
